# ONC201 selectively induces apoptosis in cutaneous T-cell lymphoma cells via activating pro-apoptotic integrated stress response and inactivating JAK/STAT and NF-κB pathways

**DOI:** 10.18632/oncotarget.18688

**Published:** 2017-06-27

**Authors:** Xiao Ni, Xiang Zhang, Cheng-Hui Hu, Timothy Langridge, Rohinton S. Tarapore, Joshua E. Allen, Wolfgang Oster, Madeleine Duvic

**Affiliations:** ^1^ Department of Dermatology, The University of Texas MD Anderson Cancer Center, Houston, TX, USA; ^2^ Oncoceutics, Inc., Philadelphia, PA, USA

**Keywords:** NHL, ONC201, TIC10, TRAIL, cancer

## Abstract

Cutaneous T-cell lymphomas (CTCLs) are extremely symptomatic and still incurable, and more effective and less toxic therapies are urgently needed. ONC201, an imipridone compound, has shown efficacy in pre-clinical studies in multiple advanced cancers. This study was to evaluate the anti-tumor activity of ONC201 on CTCL cells. The effect of ONC201 on the cell growth and apoptosis were evaluated in CTCL cell lines (n=8) and primary CD4^+^ malignant T cells isolated from CTCL patients (n=5). ONC201 showed a time-dependent cell growth inhibition in all treated cell lines with a concentration range of 1.25-10.0 μM. ONC201 also induced apoptosis in tested cells with a narrow concentration range of 2.5-10.0 μM, evidenced by increased Annexin V^+^ cells, accompanied by accumulated sub-G1 portions. ONC201 only induced apoptosis in CD4^+^ malignant T cells, not in normal CD4^+^ T cells. The activating transcription factor 4 (ATF4), a hallmark of integrated stress response, was upregulated in response to ONC201 whereas Akt was downregulated. In addition, molecules in JAK/STAT and NF-κB pathways, as well as IL-32β, were downregulated following ONC201 treatment. Thus, ONC201 exerts a potent and selective anti-tumor effect on CTCL cells. Its efficacy may involve activating integrated stress response through ATF4 and inactivating JAK/STAT and NF-κB pathways.

## INTRODUCTION

Cutaneous T-cell lymphomas (CTCLs) are a heterogeneous group of extranodal non-Hodgkin’s lymphomas. They are characterized by skin-homing malignant clonal T-lymphocytes. Mycosis fungoides (MF) and Sézary syndrome (SS) are two most common forms of CTCLs. MF can be chronic and indolent or progress to involve the blood, lymph nodes, and other internal organs [1]. SS is characterized by erythroderma and the presence of Sézary cells in the blood, which are immunophenotypically CD4^+^CD26^-^ or CD4^+^CD7^-^ T cells [2]. There are currently limited treatment options for patients with advanced CTCL, and approved therapies have response rates of around 30% [3, 4]. The currently available agents are expensive and have toxicity including immunosuppression predisposing to infections. Disease recurrence and therapy resistance are common. Thus, there remains an unmet need for novel and safe therapies to treat CTCL.

ONC201 or TIC10 is a chemical compound referred to as 7-benzyl-4-(2-methylbenzyl)-1,2,6,7,8,9-hexahydroimidazo[1,2-a]pyrido[3,4-e]pyrimidin-5(4H)-one. It is the first-in-class member of the imipridone class of anti-cancer compounds and a highly selective G protein-coupled receptor (GPCR) antagonist [5]. This oral small molecule is currently in clinical trials for advanced cancers [5]. Although it is known that ONC201 induces apoptosis in refractory tumor cells in a p53-independent manner, its other potential mechanisms of action that lead to anti-tumor activity are still under investigation [6, 7]. A prior report on the mechanism (s) of action of ONC201 in preclinical solid tumor models implicated a late stage inactivation of Akt and ERK leading to Foxo3a-mediated induction of TRAIL and its pro-apoptotic receptor DR5 [7]. Recent studies have also implicated the integrated stress response (ISR) as an early stage mechanism of ONC201 that may lead to its previously observed downstream anti-cancer effects [8]. ONC201 activates the integrated stress response that attenuates protein translation and upregulates activating transcription factor 4 (ATF4), which causes induction of genes that lead to apoptosis.

The signal transducers and activators of transcription (STAT) family members, such as STAT3, are commonly activated in CTCL [9]. STATs can be phosphorylated by one of four upstream Janus kinases (JAKs) following cytokine stimulation. Upon nuclear translocation, phosphorylated STAT3 (pSTAT3) directly regulates expression of key target genes, including cell cycle genes (Cyclin D and myc), regulators of apoptosis (BCL-2/BAX), cytokines (e.g. IL-5 and IL-13), and suppressors of cytokine signaling (SOCS3) that work together to promote carcinogenesis [10-13]. Constitutive activation of STAT1, STAT3 and STAT5 has been observed in both early and late stages of CTCL [9]. In addition, STAT3 indirectly regulates gene expression by inducing DNA methyltransferase 1 (DNMT1), thereby promoting the epigenetic silencing of tumor suppressor genes [14]. Thus, constitutively active STAT3 can increase survival and resistance to apoptosis in malignant T cells in CTCL.

Meanwhile, the dysregulated nuclear factor κB (NF-κB) pathway has also been implicated in CTCL. NF-κB is a key transcriptional regulator of cytokines controlling cell survival, differentiation, proliferation, angiogenesis, metastasis, and inflammatory responses [15]. In early stages of CTCL, autocrine tumor necrosis factor alpha (TNFα) expression increases NF-κB activation that leads to cellular proliferation and resistance to apoptosis [16, 17]. In addition to TNFα, the epidermis in patients with CTCL contains increased levels of NF-κB-dependent pro-inflammatory cytokines IL-1β, IL-8, IL-17, and IL-32 suggesting a role for these cytokines in the pathogenesis of CTCL [18-21]. Increased NF-κB activity in CTCL is also responsible for increased resistance to apoptosis by up-regulating the anti-apoptotic cellular inhibitor of apoptosis proteins (cIAP) and BCL-2 [22]. Thus, NF-κB plays a key role in CTCL by promoting inflammation and by inhibiting apoptosis.

It is reported that STAT3 and NF-κB often cooperate to promote the development and progression of solid cancers [23]. Both NF-κB and STAT3 are rapidly activated in response to various stimuli including stresses and cytokines, although they are regulated by different signaling mechanisms. Once activated, NF-κB and STAT3 can independently and/or synergistically control the expression of anti-apoptotic, pro-proliferative and immune response genes [23].

Based on the preclinical profile of ONC201 and the need for novel, safe and effective therapies for CTCL, this study was undertaken to evaluate the effect of ONC201 on CTCL cells and to understand its mechanism of action.

## RESULTS

### ONC201 inhibits cell growth in CTCL cell lines

**Figure 1:**
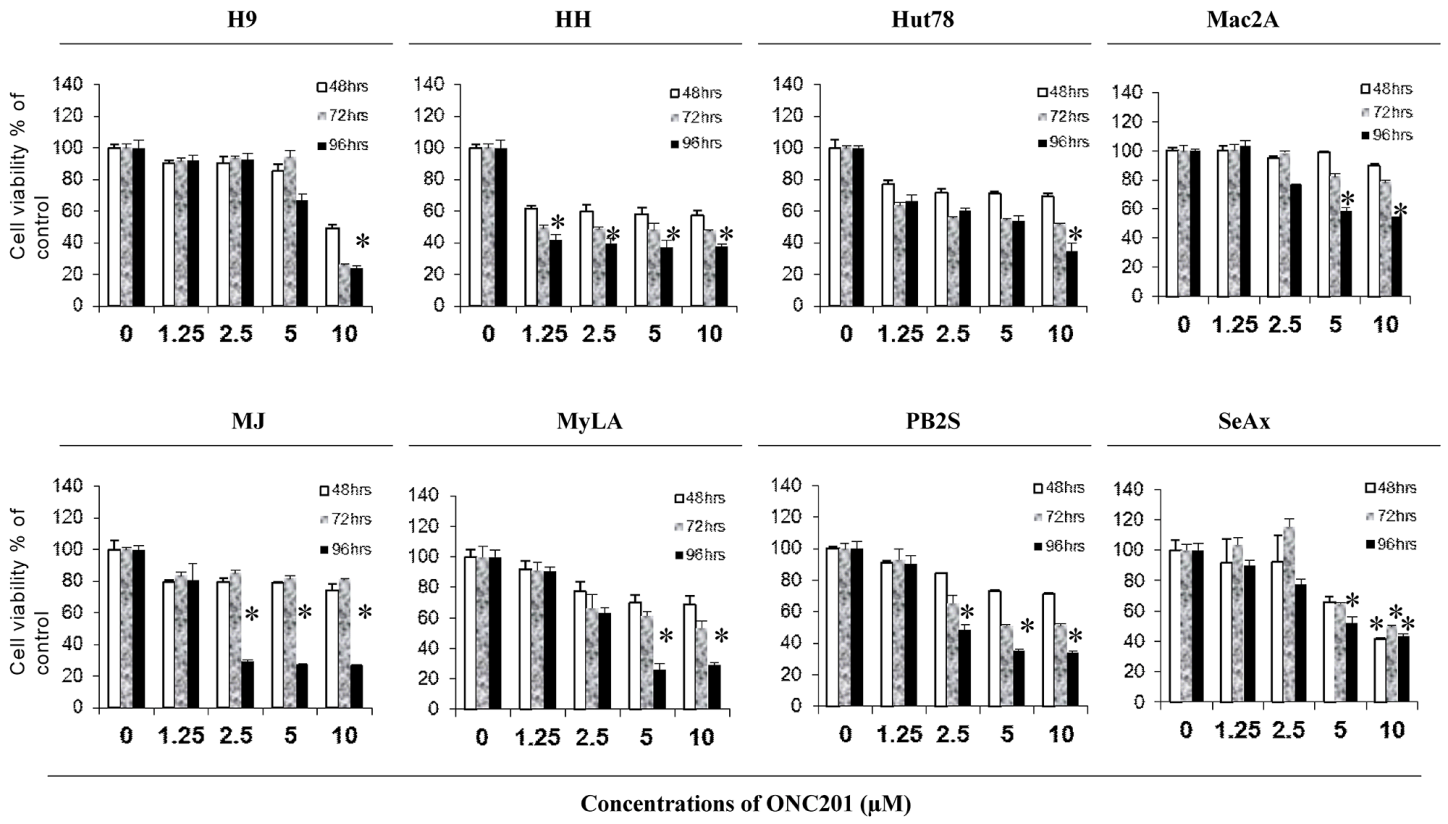
ONC201 inhibits cell growth in CTCL cell lines H9, HH, Hut78, Mac2A, MJ, MyLa, PB2S, and SeAx cells were cultured in 96-well culture plates (5×10^4/^well) with or without 1.25, 2.5, 5.0, and 10.0 μM of ONC201 for 48, 72, and 96 hrs, respectively. Cell viability was determined using CellTiter 96® Aqueous One Solution Cell Proliferation Assay (MTS). Data for 8 cell lines were presented with different doses at 3 time points (mean ± SD of triplicate determinations). *significant difference, p < 0.05.

We first assessed the anti-proliferative effect of ONC201 on CTCL cells by the CellTiter Cell Proliferation Assay (MTS). Eight CTCL cell lines were treated with ONC201 at 0, 1.25 μM, 2.5 μM, 5.0 μM, and 10.0 μM, respectively, over 48 hours (48 hrs), 72 hours (72 hrs), and 96 hours (96 hrs). As shown in Figure 1, the cell growth was inhibited in all 8 cell lines, but with different sensitivities. HH cells were the most sensitive with the cell growth inhibited from 1.25 μM of ONC201 treatment at 48 hrs (42.4%), to a maximum at 96 hrs (62.2%, p<0.05). The growth of MJ cells was inhibited at 2.5 μM, sustained at 5.0 μM or 10 μM, and reached a maximum at 96 hrs. Interestingly, the cell growth inhibition in 3 other cell lines (Mac2A, MyLA, and PB2S) was not significantly enhanced when ONC201 doses were increased from 5.0 μM to 10.0 μM. H9 cells were least sensitive, as the inhibition was only seen at 10 μM of ONC201. These results suggest that ONC201 has an anti-proliferative effect on CTCL cells, and it inhibits cell growth within a narrow concentration range from 1.25 μM to 10.0 μM in a time-dependent manner.

### ONC201 induces apoptosis in CTCL cell lines

**Figure 2:**
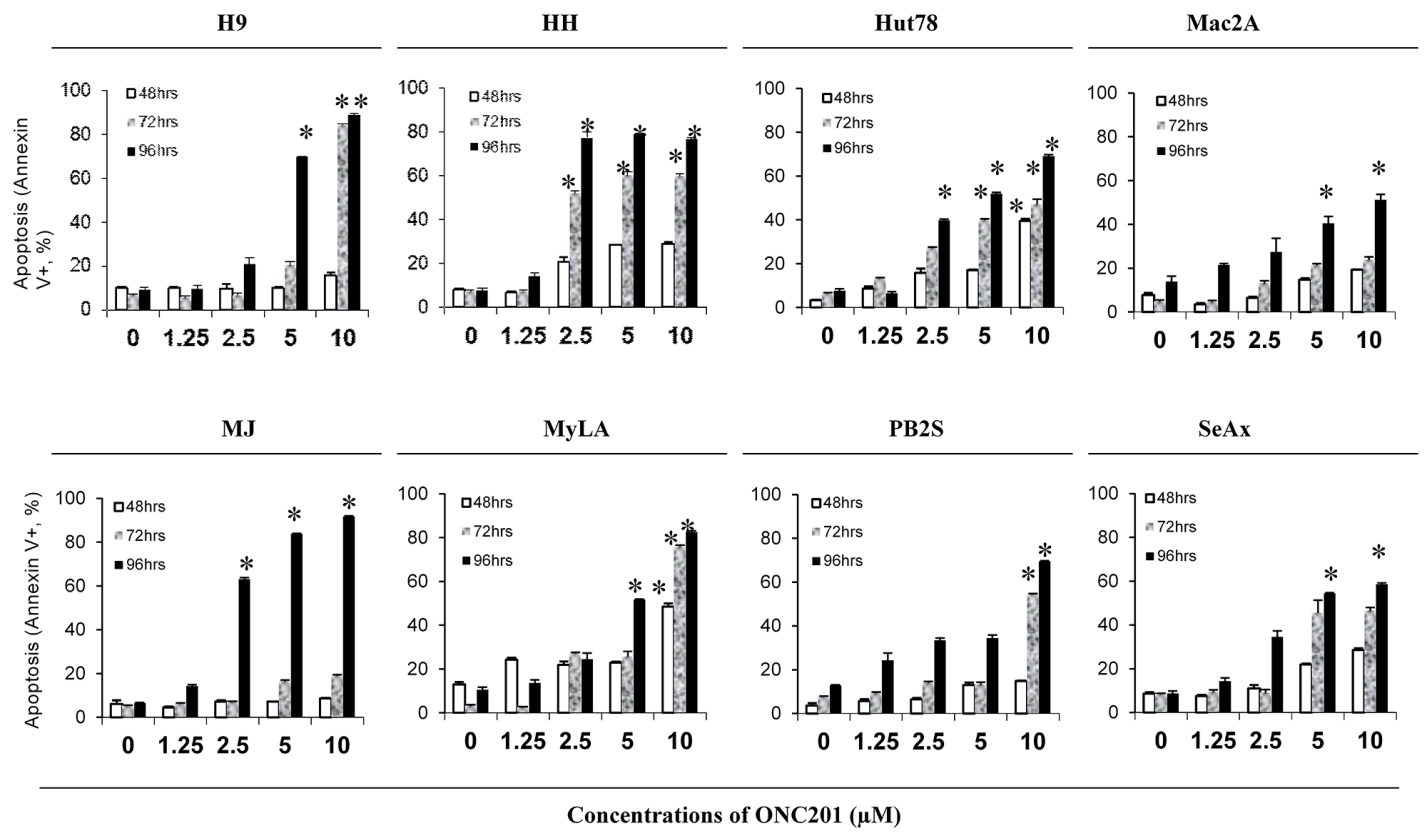
ONC201 induces apoptosis in CTCL cell lines H9, HH, Hut78, Mac2A, MJ, MyLa, PB2S, and SeAx cells (5×10^5/^ml) were treated with or without 1.25, 2.5, 5.0, and 10.0 μM ofONC201 for 48, 72, and 96 hrs. Apoptotic cells were assessed by flow cytometry using the Annexin V-FITC Detection Kit. Data were presented as the percentage of Annexin V^+^ cells for all 8 cell lines with different doses at 3 time points (mean ± SD of triplicate determinations). *significant difference, p < 0.05.

We next determined the pro-apoptotic effect of ONC201 on 8 CTCL cell lines treated with similar doses of ONC201 and at similar time points. The apoptotic cells were analyzed by flow cytometry using Annexin V/propidium iodide (PI) staining. ONC201 was able to induce apoptosis in all 8 cell lines at different doses (Figure 2). Again, HH cells were the most sensitive to ONC201, at concentrations starting at 2.5 μM over a longer 72-hour period. Apoptosis occurred in 47.9% of HH cells at 2.5 μM of ONC201 over 72 hours of treatment, which was increased by 7.6-fold in comparison to 1.25 μM at 72 hrs (6.3%), and was also doubled in comparison to 2.5 μM for a 48-hour treatment (20.3%, p<0.05). Hut78 and MJ cells had increased numbers of apoptotic cells at 2.5 μM, for a 96-hour of treatment, with low levels at early time points. ONC201 also induced apoptosis in 4 other cell lines (H9, Mac2A, MyLA, and SeAx cells) at a higher dose of 5.0 μM over a 96-hour of treatment. Compared to other cells, PB2S cells were the least sensitive to ONC201, and the induction of apoptosis was only observed at 10 μM. These results suggest that ONC201 induces CTCL cell apoptosis within a very narrow concentration range from 2.5 μM to 10.0 μM in a time-dependent manner.

**Figure 3:**
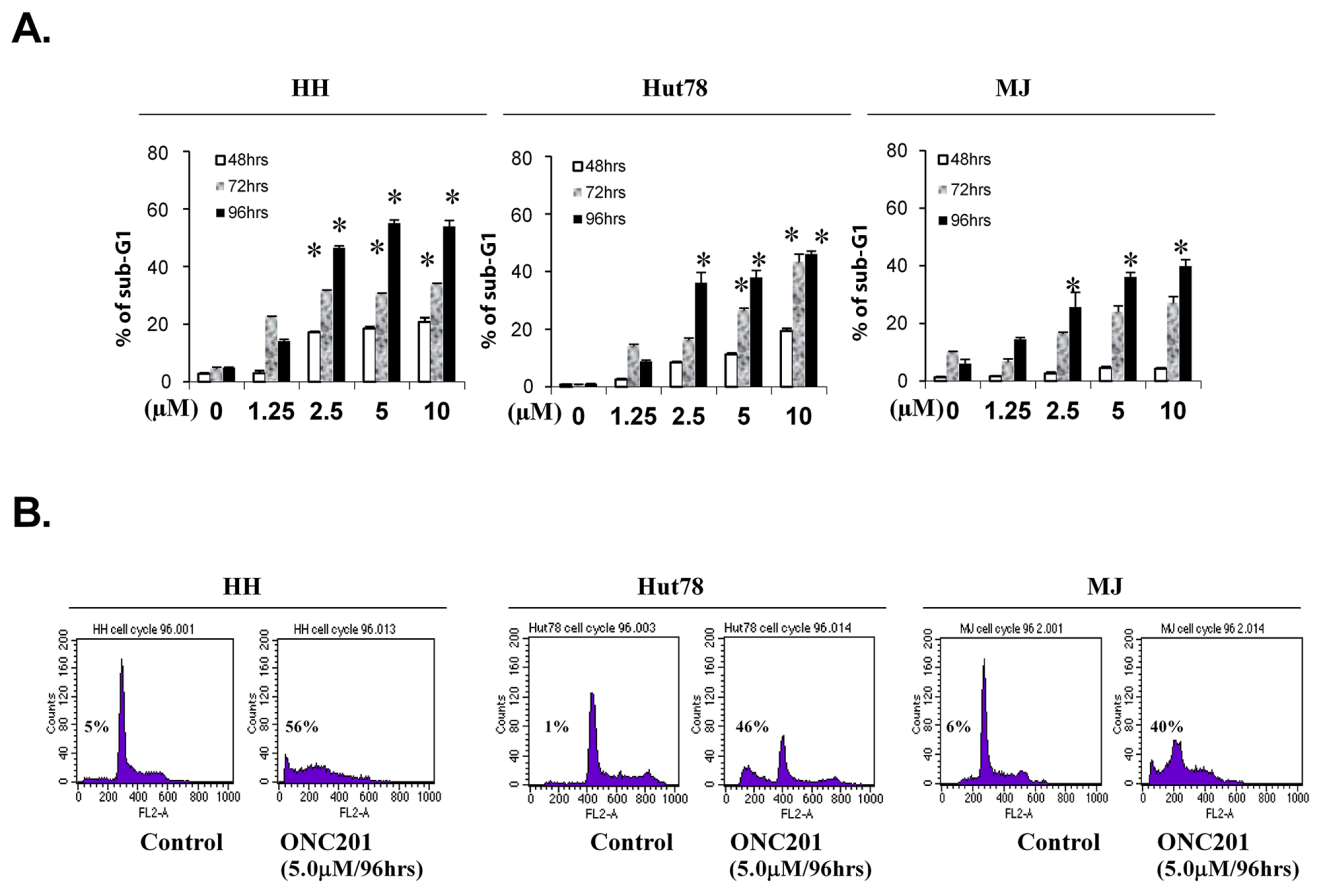
ONC201 induces accumulation of sub-G1 portions in CTCL cell lines HH, Hut78 and MJ cells (5×10^5^) were treated with or without 1.25, 2.5, 5.0, and 10.0 μM of ONC201 for 48, 72, and 96 hrs. Cells were stained with PI, and sub-G1 distributions were determined by flow cytometry. **(A)** The percentages of sub-G1 portions for HH, Hut78, and MJ cells were presented with different doses at 3 time points (mean ± SD of triplicate determinations). *significant difference, p < 0.05. **(B)** Plots for representative paired cells with or without 5.0 μM of ONC201 for 96 hrs were presented. The % of sub-G1portion in each plot was indicated.

We next examined the effect of ONC201 on cell-cycle sub-G1 and/or apoptosis by flow cytometry in 3 cell lines: SS-derived HH, Hut78 cells, and MF-derived MJ cell lines. As shown in Figure 3A&3B, sub-G1 populations were increased in all 3 cell lines in a time- and dose-dependent manner. Significant increases of sub-G1 populations were seen from 2.5 μM over 96 hrs. HH cells showed the highest sensitivity, followed by Hut78 and MJ cells, consistent with our results from Annexin V/PI staining suggesting that the cells are undergoing apoptosis.

### ONC201 selectively induces apoptosis in primary Sézary cells

**Figure 4:**
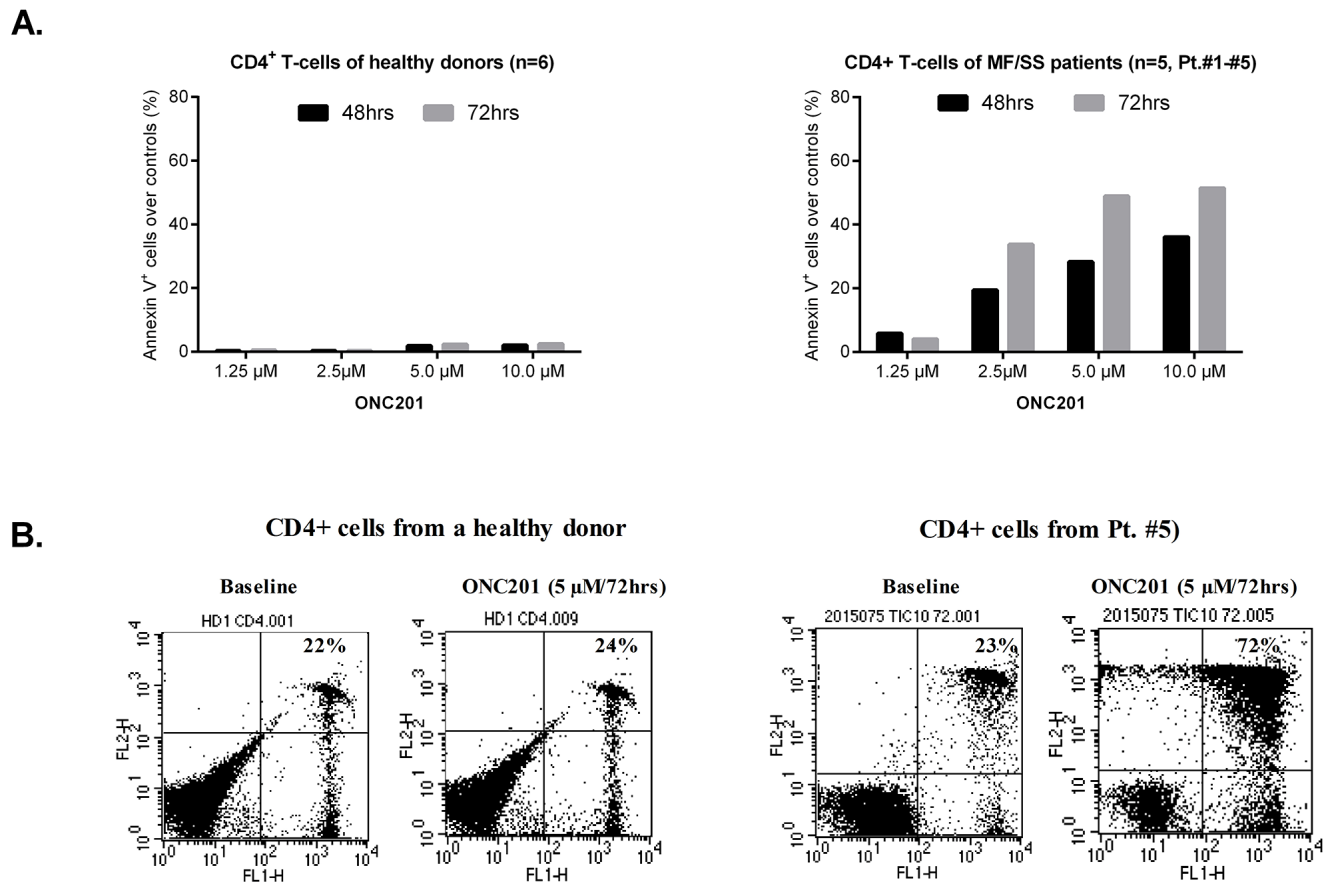
ONC201 selectively induces apoptosis in CD4^+^ malignant T cells CD4^+^ T cells (5×10^5^) from MF/SS patients and healthy donors were treated with or without 1.25, 2.5, 5.0, and 10.0 μM of ONC201 for 48 and 72 hrs. Apoptotic cells were assessed by flow cytometry using the Annexin V-FITC Detection Kit. **(A)** The percentages of Annexin V^+^ cells were presented for normal CD4^+^ T cells (mean, n=6) and malignant CD4^+^ T cells (mean, n=5; Patient #1 - #5) with different doses at two time points. **(B)** Dot plots for representative CD4^+^ T cells from a healthy donor (left) and Patient#5 with or without 5.0 μM of ONC201 for 72 hrs were presented.

One reason that ONC201 was selected as a lead compound for clinical development is its lack of toxicity in normal cells [5]. We next examined the pro-apoptotic effects of ONC201 on primary Sézary cells in comparison to normal CD4^+^ T cells from healthy donors. Primary CD4^+^ malignant T cells were isolated from the peripheral blood of 5 patients with MF/SS who had > 60% circulating CD4^+^CD26^-^ T cells of total lymphocytes (Table 1, Patient #1 - #5). Cells were incubated with or without ONC201 at concentrations ranging from 1.25 to 10 μM for 48 hrs and 72 hrs. The apoptotic cells were then assessed by flow cytometry using Annexin V/PI staining. Similar to HH and Hut78 cells, apoptotic CD4^+^ malignant T cells were dramatically increased at 2.5 μM (19.4% at 48 hrs; 33.7% at 72 hrs, n=5), in comparison to 1.25 μM (5.8% at 48 hrs; 4.1.0% at 72 hrs, n=5). There was no significant enhancement when ONC201 doses were increased from 5.0 μM to 10.0 μM (Figure 4A). In contrast, normal CD4^+^ T cells showed little response to ONC201, with an average apoptosis rate of 0.3% at 1.25 μM and 2.0% at 10 μM after a 48-hour treatment, and 0.4% to 2.4% after a 72-hour treatment (n=6). Representative flow plots for Annexin V^+^ apoptotic CD4^+^ T cells from a healthy donor (left) and Patient #5 (right) are presented in Figure 4B. As shown in Table 1, the number of CD4^+^CD26^-^ malignant T cells in the blood of Patient #5 was 3332/μL representing 79.1% of all lymphocytes in the blood. Our results suggest that ONC201 selectively induces apoptosis in primary Sézary cells but not in normal CD4^+^ T cells.

**Table 1:** Clinical demographics of patients with MF/SS

Patient #	Age/Gender*	Diagnosis**	CD4^+^CD26^-^ T-cells of total lymphocytes (%)	Absolute CD4^+^CD26^-^ T-cells (/μL)
1	80/M	SS	91.6	2800
2	72/M	MF	60.5	708
3	66/M	MF	66.9	2295
4	62/F	SS	67.5	2588
5	63/F	SS	79.1	3332
6	73/F	MF/SS	40.4	190
7	79/F	MF/SS	94.0	6612
8	59/F	MF/SS	82.6	13629

*Gender: M – male, F – female; **Diagnosis: SS – Sézary syndrome, MF – mycosis fungoides

### ONC201 activates integrated stress response through ATF4 in CTCL cells

**Figure 5:**
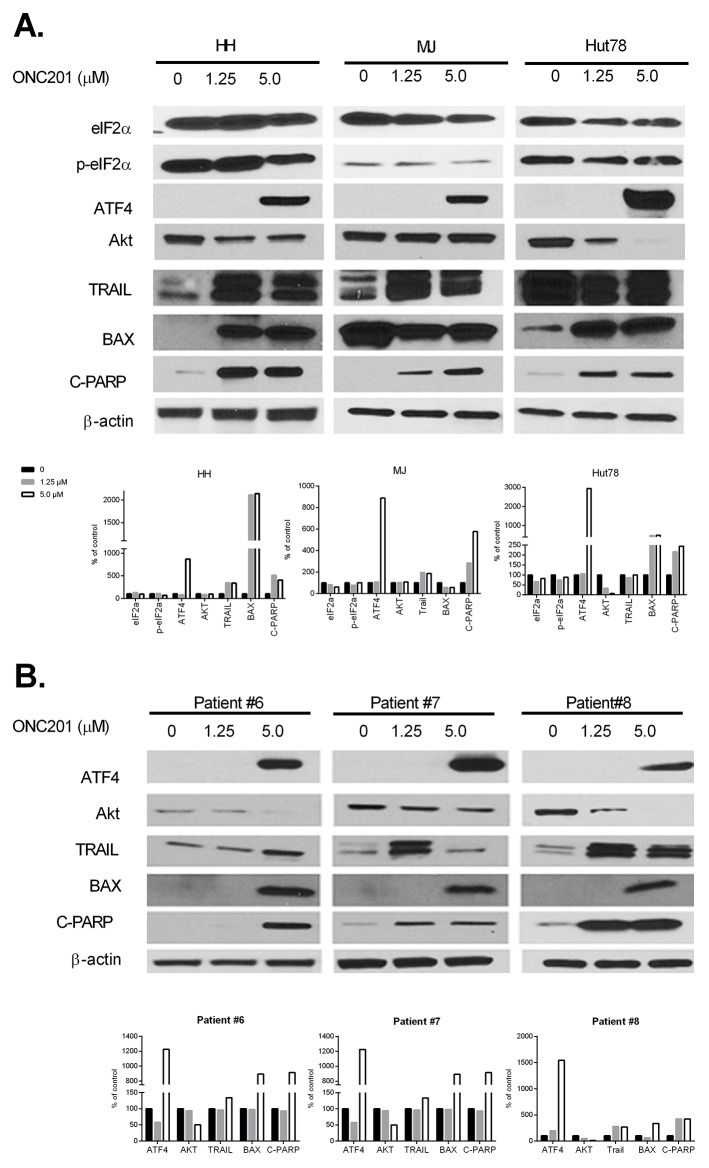
ONC201 upregulates ATF4, downregulates Akt, and induces TRAIL in CTCL cell lines and primary Sézary cells HH, Hut78, and MJ cells **(A)**, and PBMCs from 3 MF/SS patients (Patient #6 - #8) **(B)** were treated with (1.25 or 5.0 μM) or without ONC201 for 72 hrs. The protein expression of eIF2α, p-eIF2α, ATF4, Akt, TRAIL, BAX, and C-PARP were assessed by western blot. The protein level of β-actin served as housekeeping gene control. All protein levels were semi-quantified using ImageJ system (NIH), and the levels in treated cells were compared with untreated control cells which were considered as 100%.

The results above suggest a potent anti-tumor activity of ONC201 on CTCL cells, and we next investigated its mechanism(s) of action. Activation of the integrated stress response (ISR) induced by ONC201 has recently been implicated as the driver of its late downstream anti-tumor effects [5, 8, 24]. ISR activation often results in phosphorylation of eukaryotic initiation factor eIF2α,upregulation of certain transcription factors, such as activating transcription factor 4 (ATF4), an apical hallmark of the integrated stress response, and downregulation of the general protein synthesis [25]. In addition, ONC201-mediated Akt/ERK inactivation and TRAIL upregulation are implicated in colorectal cancer models [5]. We first assessed the protein expression of eIF2α, p-eIF2α, ATF4, Akt, and TRAIL by western blot in cells treated with or without 1.25 or 5.0 μM of ONC201 for 72 hrs. As expected, ONC201 induced ATF4 protein expression at 5.0 μM of ONC201 in 3 tested cell lines (Figure 5A) as well as PBMCs from 3 MF/SS patients (Figure 5B, Table 1, Patient #6-8). However, the expression of total eIF2α and p-eIF2α proteins were unchanged or slightly down-regulated in 3 treated cell lines. Akt protein levels were decreased in Hut78 cells and in PBMCs from 3 MF/SS patients starting at 1.25 μM, and enhanced at 5.0 μM. TRAIL protein expression was increased in HH and MJ cells at 1.25 and 5.0 μM of ONC201 treatment. In further support of apoptosis induced by this pharmacological manipulation, a striking increase in BAX, another pro-apoptotic protein, and caspase-mediated cleavage of poly (ADP-Ribose) polymerase (C-PARP) were documented following ONC201 treatment (Figure 5A&5B). These results suggest that ONC201 works on CTCL cells by activating ISR through inducing the expression of ATF4. Other mechanisms of action such as inactivation of Akt and induction of TRAIL, as previously reported in solid tumors [7], are also involved in mechanisms of action of ONC201 in CTCL cells. But, the induction of ATF4 in ONC201-treated CTCL cells may be independent of phosphorylation of eIF2α.

### ONC201 inactivates the JAK/STAT pathway in CTCL cells

**Figure 6:**
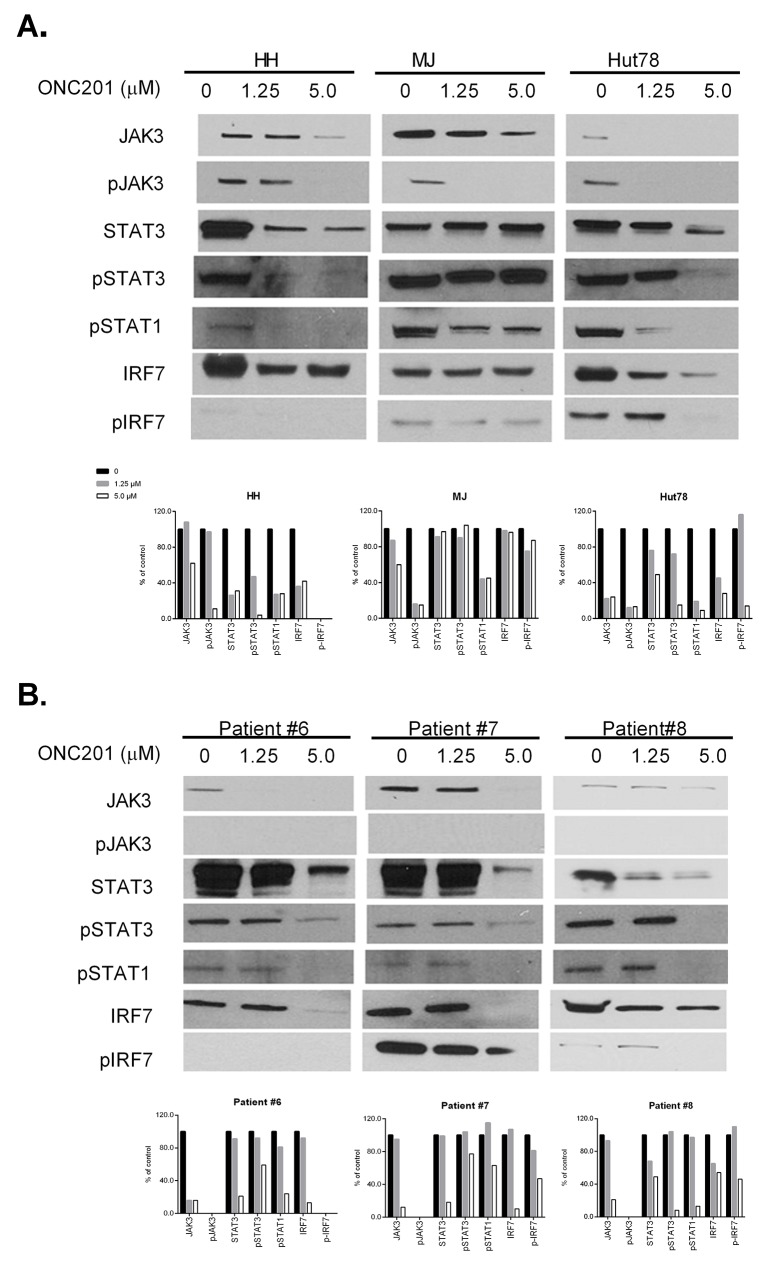
ONC201 downregulates JAK/STAT in CTCL cell lines and primary Sézary cells HH, Hut78, and MJ cells **(A)**, and PBMCs from 3 MF/SS patients (Patient #6 - #8) **(B)** were treated with (1.25 or 5.0 μM) or without ONC201 for 72 hrs. The protein expression of JAK3, pJAK3, STAT3, pSTAT3, pSTAT1, IRF7, and pIRF7 were assessed by western blot. Protein levels were semi-quantified as above, and the protein levels in treated cells were compared with untreated control cells which were considered as 100%.

Constitutive activation of the JAK/STAT pathway has been demonstrated in CTCL, and is critical for cell proliferation and survival [9, 26]. The robust activity of ONC201 on CTCL cell growth and apoptosis induction suggest that ONC201 may affect the JAK/STAT pathway in CTCL cells. Recent studies have revealed that STAT3 is negatively regulated in response to TRAIL [27]. Expression of JAK3, pJAK3, STAT3, pSTAT3, and pSTAT1 protein levels were examined by western blot in cells treated with or without ONC201. Levels of these proteins were decreased in ONC201-treated HH and Hut78 cells (Figure 6A). Their levels were also dramatically decreased in PBMCs treated with 5.0 μM of ONC201 (Figure 6B). Surprisingly, pJAK3 protein was undetectable in PBMCs from MF/SS patients, but was present in 3 cell lines. Of note, MJ cells showed a reduction of pSTAT1 protein but not STAT3 and pSTAT3 proteins as shown in Figure 6A.

In order to further explore the connection between integrated stress responses and a decreased JAK/STAT pathway, we assessed another molecule, interferon regulatory factor 7 (IRF7). ATF4 has been identified as a negative regulator of IRF7 and inhibits the transcription and phosphorylation of IRF7 [28]. As shown in Figure 6A&6B, HH and Hut78 cells and PBMCs from 3 MF/SS patients had decreased expression of IRF7 after ONC201 treatment, but not MJ cells. The expression of p-IRF7 expression was decreased in all 3 tested cell lines and PBMCs from 2 MF/SS patients (#7 and #8).

Our results support that ONC201 inactivates the JAK/STAT pathway and downregulates IRF7 in both CTCL cell lines and primary Sézary cells. IRF7 may be a connection molecule between integrated stress response and the downstream Jak3/STAT3 signaling axis.

### ONC201 downregulates the NF-κB pathway in CTCL cells

**Figure 7:**
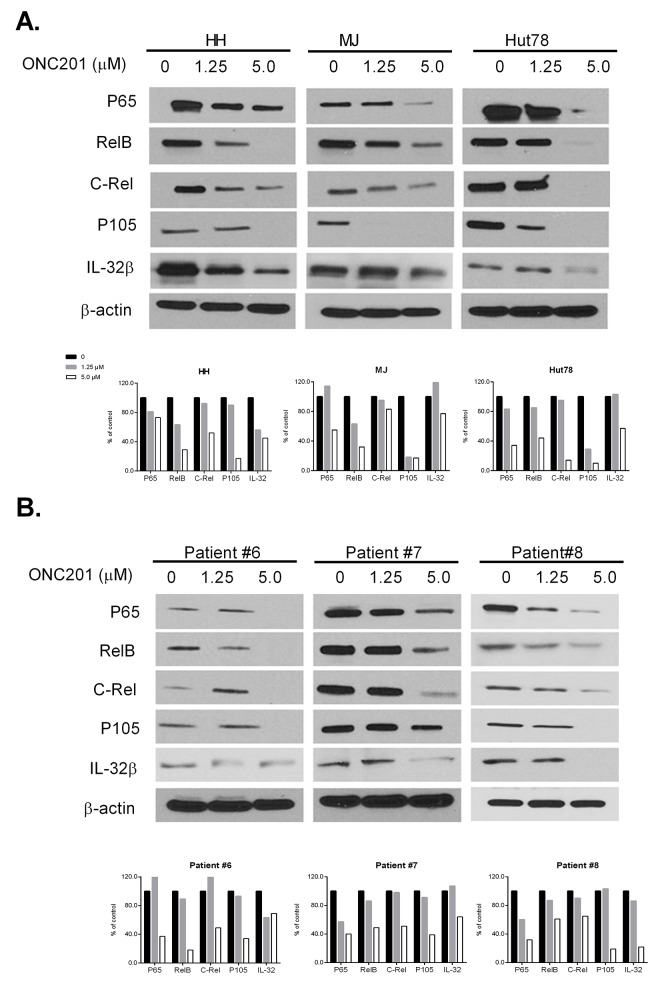
ONC201 downregulates NF-κB and IL-32 expression in CTCL cell lines and primary Sézary cells HH, Hut78, and MJ cells **(A)**, and PBMCs from 3 MF/SS patients (Patient #6 - #8) **(B)** were treated with (1.25 or 5.0 μM) or without ONC201 for 72 hrs. The protein expression of NF-κB members and IL-32β were assessed by western blot. The protein level of β-actin served as housekeeping gene control. The protein levels were semi-quantified and the levels in treated cells were compared with untreated control cells which were considered as 100%.

It is well documented that constitutive activation of the NF-κB pathway plays a role in the development of CTCL and is related to cell resistance to apoptosis [26]. Therefore, we also analyzed protein expression of NF-κB family members (p65, RelB, C-Rel and p105) by western blot in ONC201 treated cells. ONC201 significantly decreased the protein levels of all four NF-κB members in three CTCL lines (Figure 7A) and in PBMCs from 3 MF/SS patients (Figure 7B) at 5.0 μM, compared to 1.25 μM and empty controls. Our results suggest that ONC201 inactivates not only the JAK/STAT pathway, but also the NKκB pathway in CTCL cells. These effects may underlie the therapeutic effects of ONC201 on CTCL cells.

### ONC201 downregulates IL-32β expression in CTCL cells

IL-32 is known as a pro-inflammatory cytokine that is likely involved in the lymphomagenesis of CTCL [29]. We recently reported that both IL-32 transcripts and cellular IL-32β protein were highly increased in both CTCL cell lines and malignant T cells from MF/SS patients [30]. Of interest, NK-κB, NF-κB1, and STAT3 are key transcription factors regulating IL-32 gene expression. We found decreased levels of IL-32β protein in all ONC201-treated cells, in parallel with downregulation of NK-κB proteins as indicated in Figure 7A&7B.

### Down-regulation of JAK/STAT and NF-κB pathways is not transcriptionally mediated

We next addressed whether the changes in protein expression after ONC201 treatment are a consequence of a transcriptional regulation or not. The mRNA levels of most aforesaid molecules in 3 CTCL cell lines with or without ONC201 at 72 hrs were examined by real-time PCR. As summarized in Table 2, only IL-32 in Hut78 cells and STAT1 in MJ cells were slightly reduced after treatment with ONC201 for 72 hours. The mRNA levels of the rest of the molecules increased slightly in treated cell lines, especially in HH cells with a concentration at 5.0 μM. For ATF4, the mRNA levels were slightly increased at 1.25 μM, and dramatically increased by 11.8-fold in HH cells at 5.0 μM. These results suggest that the effect of ONC201 on the down-regulation of JAK/STAT and NF-κB pathways is not transcriptionally mediated. The slightly elevated mRNA levels may be a consequence of attenuation of protein translation that occurs with ISR activation.

**Table 2:** Changes in mRNA levels of related molecules in 3 CTCL cell lines treated with ONC201

	HH			MJ			HuT78		
ONC201 for 72 h	0 μM	1.25 μM	5.0 μM	0 μM	1.25 μM	5.0 μM	0 μM	1.25 μM	5.0 μM
**eIF2S1**	1.00	1.07	5.78	1.00	1.04	1.55	1.00	1.12	2.35
**ATF4**	1.00	1.78	11.80	1.00	1.23	2.17	1.00	1.26	2.08
**AKT1**	1.00	1.24	2.02	1.00	1.56	2.29	1.00	1.40	2.15
**BAX**	1.00	1.41	6.81	1.00	1.43	2.69	1.00	0.85	1.69
**JAK3**	1.00	1.79	5.76	1.00	1.56	1.69	1.00	1.20	2.25
**STAT1**	1.00	2.56	7.07	1.00	0.96	0.75	1.00	1.49	1.35
**STAT3**	1.00	1.28	4.21	1.00	1.44	1.59	1.00	1.33	2.55
**IRF7**	1.00	2.16	15.93	1.00	1.02	2.52	1.00	0.82	1.57
**NF-κB**	1.00	1.43	3.30	1.00	1.06	1.39	1.00	1.18	1.38
**IL32**	1.00	1.25	3.59	1.00	1.09	1.84	1.00	0.72	0.61

## DISCUSSION

We present pre-clinical data showing that ONC201 as a single agent demonstrated potent anti-cancer activity in CTCL cells by inhibiting cell proliferation and strongly inducing apoptosis in CTCL cell lines and primary lymphoma cells. The activity of ONC201 occurred in the low micromolar range, which is achievable *in vivo* based on prior animal experiments and results from the first-in-human trial [7, 31]. Importantly, ONC201 was more effective in primary Sézary cells and SS-derived cell lines that are more aggressive and refractory, which consistent with prior published findings [7, 32] and highlights its potential clinical utility in advanced stage patients. Our study confirms that ONC201 works on CTCL cells also by activating ISR through inducing the expression of ATF4, inactivation of Akt, and induction of TRAIL, as previously reported in solid tumors. In addition, we are first to show that ONC201 can inactivate the JAK/STAT pathway as well as the NK-κB pathway in CTCL cells.

Clinical management of MF/SS starts with skin directed therapies, then addition of retinoid or interferon, targeted therapy, and radiation. Chemotherapy is used in the setting of transformed MF or nodal involvement. We previously reported that bexarotene and histone deacetylase inhibitors (HDACi), vorinostat and romidepsin, induce apoptosis of CTCL cells *in vitro* and are also active in CTCL patients [3, 33, 34]. Patients with advanced CTCL usually develop resistance to available treatments leading to disease progression and opportunistic infections [35]. Thus, more effective and less immunosuppressive anti-cancer agents are urgently needed for advanced CTCL patients.

In accordance with prior studies in other tumor types [36], ONC201 also induced the pro-apoptotic ligand TRAIL in CTCL cells, a critical effector mechanism in the immune surveillance of cancer. Vorinostat, a HDAC inhibitor approved for CTCL [34, 37], also upregulates transcription of TRAIL [38, 39]. It is promising that ONC201 induces the same pro-apoptotic ligand as a clinically approved agent in CTCL, although the mechanism of vorinostat-mediated TRAIL gene upregulation is distinct from that of ONC201 [40].

Previous studies suggest that ONC201 activates ISR by upregulating ATF4 [5, 8, 24, 41]. ATF4 has also been identified as a negative regulator of IRF7, which is mediated by direct binding interactions in addition to inhibition of the transcription and phosphorylation of IRF7 [28]. Activation of IRF7, a master regulator of interferon gene expression, triggers the induction of IFNα/β, type I interferons, which binds to receptors to activate the JAK/STAT pathway [42]. Thus, ONC201-mediated inactivation of the JAK/STAT pathway may be a consequence of ATF4 induction that can block IRF7 activation, resulting in decreased induction of interferons and decreased subsequent activation of the JAK/STAT pathway. JAKs can phosphorylate tyrosines on receptors that ultimately stimulate the Ras signaling cascade, thereby activating Akt and ERK at a downstream level [42]. Prior studies with ONC201 in solid tumors have demonstrated a late stage inactivation of Akt and ERK, which results in less phosphorylated Foxo3a that can then enter the nucleus to upregulate TRAIL and other target genes. Thus, disruption of the JAK/STAT pathway by ONC201 may contribute to decreased activation of Akt and ERK as a late stage event of ONC201-induced signaling. However, the experiments to isolate the role of each target/pathway need be performed to understand the importance of each down-regulated pathway (NF-κB, JAK/STAT, and Akt) on anti-tumor effects by ONC201 on CTCL cells.

ISR activation often results in phosphorylation of eIF2α and upregulation of certain transcription factors, such as ATF4. However, in this study, we only found an induction of ATF4 in ONC201-treated CTCL cells, but no induction of eIF2α and p-eIF2α proteins in ONC201-treated CTCL cells. In fact, while eIF2α-dependent ATF4 induction has been shown with ONC201 in several settings, there have been a few exceptions where we have seen eIF2-independent ATF4 upregulation [8]. Ishizawa et al found that ONC201 induced an atypical integrated stress response in mantle cell lymphoma and acute myeloid leukemia cells, and the increase of ATF4 in ONC201-treated hematopoietic cells promoted apoptosis and did not depend on increased phosphorylation of eIF2α [8]. Thus, the induction of ATF4 in ONC201-treated CTCL cells may be independent of phosphorylation of eIF2α. Recent studies report that ONC201 also antagonizes the dopamine receptor D2 (DRD2)-like subfamily of G protein-coupled receptors (GPCRs) [43, 44]. DRD2 antagonism increases cyclic AMP and subsequently activates cAMP response element-binding protein (CREB). CREB has a positively regulating binding site on the ATF4 promoter [45]. Future studies on DRD2 and the cAMP signal pathway in ONC201-treated CTCL cells may help us disclose the mechanism(s) of action of ONC201 on CTCL.

The JAK/STAT pathway has been successfully targeted with small molecule inhibitors. Clinical trials are ongoing for Pacritinib and AZD9150 for the treatment of refractory colorectal cancer (NCT02277093) and hepatocellular carcinoma/non-Hodgkin’s lymphoma (NCT01563302) [46]. In CTCL, preclinical studies have shown that the pharmacological inhibition of STAT3 promotes apoptosis [47, 48]. For example, the potent JAK/STAT3 inhibitor, cucurbitacin I, decreases STAT3 phosphorylation, resulting in apoptosis of Sézary cells [49, 50]. Further studies in CTCL could explore the combinatorial efficacy of ONC201, which was recently revealed to be broadly synergistic [51].

In the past year, our group and others published findings of heterogeneous driver mutations and loss or gain of chromosomal regions using whole exome sequencing with or without RNA-seq that should ultimately advance our understanding of CTCLs [52-54]. Loss of tumor suppressor genes is the most common findings, particularly loss of chromosome 10q region [52, 55, 56], which encodes tumor suppressor genes such as PTEN, MXI1, and DMBT1, and loss of 17p region, encoding p53 and CRK [55]. Since mutations in p53 are among the most common mutations found in SS patients, ONC201 may be ideal for SS patients because its anti-tumor activity is independent of p53 [5].

ONC201 shows limited effects of anti-proliferation and apoptosis induction at 48 hours in treated CTCL cell lines, and the maximum activity was observed at 96 hours after single dose incubation. It is noticed that ONC201 has a short half-life, about 11 hours in humans [44], but its pharmacodynamics (PD) typically lasted several hours after single-dose administration that persists far beyond its pharmacokinetics (PK) [5]. This disconnect between PK and PD is now considering in designing combination therapy. ONC201 administered one to two days prior to another anti-cancer drug has demonstrated synergistic efficacy in preclinical models [8, 57].

In summary, ONC201 appears to be an active agent that impacts key signaling pathways in aggressive and refractory CTCL preclinical models through an apparently unique mechanism. The confirmation of robust activity in refractory patient samples is a good indicator that the anti-cancer activity of ONC201 will translate to the clinic. This notion is further supported by the fact that ONC201 modulates signaling pathways that have been successfully targeted in CTCL and other malignancies. ONC201 exhibits an uncommonly benign safety profile in preclinical settings and in early clinical trial experience. Advanced CTCL patients with limited tolerance for toxic regimens may be a suitable indication for ONC201 to offer therapeutic value. Another advantage of ONC201 is that it can be given as a pill taken once weekly. Along with the preclinical efficacy evidence and absence of toxicity, the coordinate disruption of key signaling pathways by ONC201 that are of highly significant relevance in the pathophysiology of CTCL warrants the clinical examination of ONC201 in advanced CTCL.

## MATERIALS AND METHODS

### Reagents

ONC201 was obtained from Oncoceutics, Inc. (Philadelphia, PA), dissolved in DMSO to a stock concentration of 20 mM, and stored at -20 °C for further use. Serial dilutions (1.25, 2.5, 5.0 and 10 μM) were made in RPMI-1640 Medium (Sigma-Aldrich, St Louis, MO) for this study.

### Cells and cell culture

Human CTCL cell lines derived from SS (HH, Hut78) and MF (MJ) were purchased from ATCC (American Type Culture Collection, Rockville, MD). PB2S, H9, SeAx, MyLa, and Mac2A, were kindly provided by Dr. Ivan Litvinov (Department of Medicine, McGill University, Canada). The peripheral blood was collected from 6 healthy donors and from 8 MF/SS patients who had high counts of blood CD4^+^CD26^-^ malignant T-cells (Table 1). Peripheral blood mononuclear cells (PBMCs) were isolated, and followed by CD4^+^ T cell selection using CD4^+^ T cell isolation kit (Miltenyi Biotec, San Diego, CA). The purity was analyzed by flow cytometry. This study was conducted according to the Declaration of Helsinki Principles. The study was approved by the institutional review board of the University of Texas MD Anderson Cancer Center. All CTCL cells were grown in RPMI-1640 Medium (Sigma-Aldrich, St. Louis, MO) supplemented with 10% heat-inactivated FBS (Atlanta Biologicals, Norcross, GA), 2mM HEPES, and 1% penicillin-streptomycin in a humidified atmosphere with 5.0% CO_2_ at 37°C.

### Cell proliferation assay

Cells were seeded in 96-well plates at a density of 5 × 10^4^ cells/well in 200 μL of complete medium, and incubated with (1.25, 2.5, 5.0 and 10 μM) or without ONC201 treatment for 48, 72, or 96 hrs, respectively. The cell viability was then determined using the CellTiter 96 Aqueous One Solution Cell Proliferation Assay (MTS) (Promega, Madison, WI) as previously described [33]. Absorbance was measured at 490 nm using the μQuant plate reader (Biotek, Winooski, VT). Experiments were performed in triplicate.

### Apoptosis analysis by flow cytometry

Cells were incubated at 5 × 10^5^ cells/mL with (1.25, 2.5, 5.0, and 10 μM) or without ONC201 treatment for 48, 72 or 96 hrs, and then harvested. The apoptotic cells were analyzed by flow cytometry using the Annexin V-FITC Detection Kit I (BD Pharmingen, San Diego, CA) as previously described [33].

### Cell cycle analysis by flow cytometry

Cells were incubated at 5 × 10^5^ cells/mL with 1.25, 2.5, 5.0, and 10 μM or without ONC201 treatment for 48, 72 or 96 hrs, and then harvested. After washing with cold phosphate-buffered saline (PBS), cells were fixed in cold 100% ethanol and stored at -20°C for 1 hour. They were then treated with DNase-free RNase (Roche Diagnostics, Basel, Switzerland) and stained with 50 μg per mL of propidium iodide (PI) (Sigma-Aldrich, St. Louis, MO). Distribution of cell cycle phases by varying DNA content was determined with a FACSCalibur flow cytometer (Becton Dickinson). Analyses of cell cycle distribution, including of sub-G1 populations, were performed as previously described [33].

### Western blot and semi-quantification analysis

The cellular proteins (5 or 10 μg) extracted from treated cells were subjected to 4-20% Mini-Protean TGX gel (Bio-Rad, Hercules, CA) electrophoresis and transferred onto nitrocellulose membranes (Whatman GmbH, Dassel, Germany). The membranes were blocked in 5.0% milk in TBST (50 mM Tris pH 7.5, 150 mM NaCl, 0.05% Tween 20) for 1 hour at room temperature, then incubated with primary antibodies overnight at 4 °C in 5.0% milk in TBST. Then membranes were incubated with 1:2,000 peroxidase-conjugated anti-mouse or anti-rabbit secondary antibodies (Cell Signaling, Beverly, MA) for 1 hour at room temperature. The primary antibodies and dilutions used in this study were listed as follows: eIF2α 1:1000, p-eIF2 1:1000, ATF4 1:2000, AKT 1:2000, TRAIL1:2000, BAX1:2000, cleaved-PARP 1:2000, JAK3 1:1000, pJAK3 1:1000, STAT3 1:2000, pSTAT13 1:2000, pSTAT1 1:2000, IRF7 1:2000, p-IRF7 1:1000, NF-κB p65 1:2000, RelB 1:1000, c-Rel 1:1000, IL-32β 1:1000, β-Actin 1:5000 (Cell Signaling, Beverly, MA). Protein bands were visualized using the Super Signal West Pico Chemiluminescence Substrate kit (Thermo, Rockford, IL). Equivalent loading of proteins in each well was confirmed by β-actin and Ponceau staining. For semi-quantification of protein expression, target bands were scanned. Then, scanned images were converted to 8-bit format images using ImageJ software (NIH). Density of each band was used for further calculation. The levels of each protein in treated cells were compared with untreated control cells which were considered as 100% [26].

### Quantitative real-time PCR for mRNA expression

Total RNA was extracted by RNeasy Mini kit (Qiagen, Valencia, CA) from HH, MJ, and Hut78 cells with ONC201 treatment at 0, 1.25 μM, and 5.0 μM for 48 hours and 72 hours. First strand cDNA was synthesized from 1000 ng of total RNA with an oligo (dT) 12–18 primer using Superscript IV reverse transcriptase (Life Technologies Inc., Gaithersburg, MD). Pre-formulated TaqMan primers and probes for eIF2S1 (Hs00187953_m1), ATF4 (Hs00909569_m1), Akt1 (Hs00178289_m1), BAX (Hs00180269_m1), JAK3 (Hs00354555_m1), STAT1 (Hs01013996_m1), STAT3 (Hs00374280_m1), IRF7 (Hs01014809_m1), NFKB1 (Hs00765730_m1), IL-32(Hs00992441_m1), were used. Glyceraldehye-3-phosphate dehydrogenase (GAPDH, Hs02786624-g1) was used as endogenous control gene. Quantitative PCR was run in the ABI Prism 7000 Sequence Detection System using the default protocol by the manufacturer (Applied Biosystems, Foster City, CA). The relative levels of mRNA expression were quantitated based on the Ct value and then normalized to GAPDH. Relative fold changes were finally calculated [58].

### Statistical analysis

The differences in cell viability, apoptosis, and sub-G1 of cell cycle in ONC201 treated cells were compared with the untreated cells or vehicle controls. Statistical significance was determined by Student’s or paired t-test. The minimum level of significance was p<0.05. Experiments were repeated twice and carried out in triplicate.

## Abbreviations

GPCR: G protein-coupled receptor
DRD2: dopamine receptor D2
CREB: cAMP response element-binding protein
eIF2α: eukaryotic initiation factor 2α
ATF4: activating transcription factor 4
TRAIL: TNF-related apoptosis-inducing ligand
DR5: death receptor 5
TNF: tumor necrosis family
IRF7: interferon regulatory factor 7
CTCL: cutaneous T-cell lymphoma
